# Gender difference and sex hormone production in rodent renal ischemia reperfusion injury and repair

**DOI:** 10.1186/1476-9255-8-14

**Published:** 2011-06-09

**Authors:** René Robert, Daniel Aiham Ghazali, Frédéric Favreau, Gérard Mauco, Thierry Hauet, Jean-Michel Goujon

**Affiliations:** 1 CHU Poitiers, Service de Réanimation Médicale Poitiers, F-86000, France; 2 CHU Poitiers, Service des Urgences, Poitiers, F-86000, France; 3 CHU Poitiers, Service de Biochimie, Poitiers, F-86000, France; 4 CHU Poitiers, Service d'Anatomopathologie, Poitiers, F-86000, France; 5 Inserm U927, Poitiers, F-86000, France; Université de Poitiers, Faculté de Médecine et de Pharmacie, Poitiers, F-86000, France

## Abstract

**Background:**

Several lines of evidence suggest a protective effect of female sex hormones in several organs subjected to ischemia-reperfusion injury. The aim of the study was to investigate sex hormone production in male rats after a renal ischemia-reperfusion sequence and analyze the influence of gender differences on tissue remodelling during the recovery process.

**Method:**

Age-matched sexually mature male and female rats were subjected to 60 min of renal unilateral ischemia by pedicle clamping with contralateral nephrectomy and followed for 1 or 5 days after reperfusion. Plasma creatinine, systemic testosterone, progesterone and estradiol levels were determined. Tubular injury, cell proliferation and inflammation, were evaluated as well as proliferating cell nuclear antigen, vimentin and translocator protein (TSPO) expressions by immunohistochemistry.

**Results:**

After 1 and 5 days of reperfusion, plasma creatinine was significantly higher in males than in females, supporting the high mortality in this group. After reperfusion, plasma testosterone levels decreased whereas estradiol significantly increased in male rats. Alterations of renal function, associated with tubular injury and inflammation persisted during the 5 days post-ischemia-reperfusion, and a significant improvement was observed in females at 5 days of reperfusion. Proliferating cell nuclear antigen and vimentin expression were upregulated in kidneys from males and attenuated in females, in parallel to injury development. TSPO expression was transiently increased in proximal tubules in male rats.

**Conclusions:**

After ischemia, renal function recovery and tissue injury is gender-dependent. These differences are associated with a modulation of sex hormone production and a modification of tissue remodeling and proliferative cell processes.

## Background

Several lines of evidence suggest that both humoral and cell-mediated immunity are more active in females than in males [1]. Sex steroid hormones could play a pivotal role [2,3] in this process, and act as regulators of inflammatory processes. Indeed, numerous inflammatory cell functions, such as neutrophil chemoattractant generation, phagocytic responses of neutrophils [4], and nitric oxide production by alveolar macrophages [5] show gender differences.

Ischemia-reperfusion injury (IRI) is known to exacerbate a pro-inflammatory milieu. The influence of gender on IRI has been mainly studied in cardiac ischemia and it is now established that female steroid hormones play a protective role [6-8]. In liver, it was reported that female mice were protected from liver ischemia-reperfusion compared to males, linked to an estrogen-dependent mechanism [9,10]. In a renal ischemia-reperfusion model, the mortality rate was significantly greater in male rodents [11,12]. Orchidectomy significantly improved male survival [11] and testosterone may increase kidney ischemic injury in mice [13]. These results suggest a protective effect of female hormones, and a detrimental effect of male hormones. However, although sex hormones could play an important role, the direct gender influence has not been fully demonstrated in renal ischemia-reperfusion models and the mechanisms involved remain to be elucidated.

We have previously demonstrated, in a rodent kidney ischemia-reperfusion model, that extensive renal tissue injury such as proximal tubule necrosis and outer medulla congestion occurred during the reperfusion phase following 60 min of ischemia [14]. In these conditions, tissue remodelling is determined by the severity of ischemia as we have previously shown in a pig model of renal ischemia reperfusion [15]. Tissular regeneration could be a pivotal key of organ outcome, limiting or counteracting injury development. Different markers of tissue remodelling or regeneration have been used in renal tissue such as vimentin, alpha-smooth muscle actin (α-SMA), proliferating cell nuclear antigen (PCNA) or translocator protein (TSPO) expressions. TSPO, formerly known as the peripheral-type benzodiazepine receptor [16], is a widely distributed transmembrane protein that is localized mainly in the outer mitochondrial membrane. Many functions are associated directly or indirectly with TSPO, including the regulation of cholesterol transport and steroid hormones synthesis, porphyrin transport and heme synthesis, apoptosis, cell proliferation, anion transport, regulation of mitochondrial functions and immunomodulation [16,17]. TSPO expression has been shown to be modulated by renal ischemia-reperfusion injury, in particular during the repair process [18,19]. Under these conditions, IRI could affect TSPO expression and sex hormone production respectively, modulating cellular response.

The aim of this study was to investigate sex hormone production in male rats subjected to renal ischemia-reperfusion, and analyze the influence of gender differences on tissue remodelling markers, particularly on cell proliferation processes and TSPO expression in the early phase of reperfusion.

## Material and methods

### Ischemia-reperfusion procedure

Age-matched (4 to 5 month-old) male and female Sprague-Dawley rats weighting 220-380 g were studied (Depré, France). All animals were sexually mature. The surgical and experimental protocols were performed in accordance with the French Ministry of Agriculture for the use and care of laboratory animals. The protocol was approved by the local Ethical Committee for animal study and referenced with the number 0206.

### Experimental Design

Eight rats (4 males and 4 females) were considered as controls and received only anesthesia and sham surgery. Ischemia-reperfusion experiments were performed in 40 rats, including 21 male and 19 female rats. On the day of experiment, animals were anesthetized with an intramuscular injection of a mix of medetomidine chlorhydrate 0.4 mg/kg (Pfizer, Orsay, France) and ketamine chlorhydrate 25 mg/kg (Pfizer, Orsay, France). In all rats, after a middle laparotomy incision, the right kidney was removed, mimicking the clinical conditions of nephron mass reduction. Ischemia was induced in the contralateral kidney by clamping the renal artery. After 60 minutes, the non traumatic clamp was removed inducing renal reperfusion and the abdomen wall was closed. Blood samples were collected at different time intervals for plasma creatinine and hormone levels determination. Plasma creatinine concentrations were determined using a Hitachi 917 automatic analyzer (Roche Diagnostic, Meylan, France). At the time of sacrifice, rats were anesthetized using the protocol described above and left kidney was removed. Three end-points were studied for male and female rats: D0 (kidney removed immediately after the 60-min ischemia without reperfusion, n = 6 males and 5 females), D1 (kidney removed after the 60-min ischemia and 24 hours of reperfusion, n = 7 males and 6 females) and D5 (kidney removed after the 60-min ischemia and 5 days of reperfusion, n = 8 males and 8 females). Two female rats died immediately after anesthesia, before undergoing ischemia-reperfusion experiment, and were not considered in data analysis.

### Histochemical and immunohistochemical studies

Samples were fixed in Dubosq-brazil, 10% formalin in 0.01 mM phosphate buffer (pH 7.4), and embedded in paraffin. Then, periodic acid-Schiff staining was applied for histochemical analysis. A pathologist performed analyses in a blinded fashion using standard histopathological method. Two basic histological patterns typical of proximal tubular injury: loss of brush border and cell detachment, were graded according to a published semi-quantitative score using a 5-point scale as follows: 0, no abnormalities, 1, mild lesions affecting less than 25% of kidney section; 2 lesions affecting 25% to 50% of kidney section; 3 lesions affecting 50% to 75% of kidney section; and 4 lesions affecting more than 75% of kidney section [20].

We assessed cell proliferation and dedifferentiation to evaluate the time course of proximal tubule cell regeneration after IRI. We used specific antibodies against PCNA (1:100, Neomarkers Data Sheet, Lab Vision, UK), and vimentin (1/100, Dakopatts, Denmark) as markers respectively of cell proliferation and cell dedifferentiation. We established the percentage of vimentin-positive proximal tubule cells and the proliferation index (PI) as the percentage of PCNA-positive nuclei, in ten high-power fields (x400). Patterns of TSPO expression in kidney, was analysed using a rabbit polyclonal antibody anti-mouse TSPO (1/100, a gift from Vassilios Papadopoulos Department of Biochemistry and Molecular Biology, Georgetown University, Washington DC, USA) directed against amino acid sequences conserved across species [21]. TSPO immunostaining was graded using an arbitrary scale: 0: No staining; 1: Faint staining; 2: Moderate staining; and 3: Strong staining. Infiltrated neutrophils and T lymphocytes, indicating innate and adaptative immunity, were detected respectively using anti-Ly6G (GR-1, 1/1000, eBiosciences, San Diego, USA) and anti-CD3 (1/200, Dakopatts) antibodies. The number of Ly6G and CD3 positive labeled cells per surface areas (10^4 ^µm^2^) were counted on ten different kidney tissue sections.

### Male sex hormone quantification

Testosterone, progesterone and estradiol levels were evaluated in systemic blood by double antibody radioimmunoassay (DA RIA; Diagnostic Systems Laboratories, Inc (DSL), Texas, USA).

### Statistical Analysis

Data were expressed as mean ± standard error of the mean (SEM). Continuous values were compared using Student's t test. The Mann-Whitney-Wilcoxon signed-ranks test was used for non Gaussian distribution. Statistical significance was defined as p <0.05.

## Results

### Effect of ischemia-reperfusion on renal functional parameters and kidney outcome

Plasma creatinine levels (table 1) were increased with similar degree in males and females at the end of the ischemic period (D0) (96.1 ± 5.6 vs. 118.0 ± 6.9). After 1 and 5 days of reperfusion, plasma creatinine was significantly higher in males than in females (D1, 345.0 ± 6.6 vs. 200.5 ± 6.6; D5, 511.7 ± 6.4 vs. 253.0 ± 6.6 µmol/L; p <0.05 and <0.01 respectively). Plasma creatinine concentrations in each group before ischemia-reperfusion sequences did not show statistical difference (data not shown). These results were supported by survival analysis demonstrating a poor survival rate in males compared to females (at day 5, 5 out of 8 males died whereas only 3 out of 8 females died).

**Table 1:** Male and female rat plasma creatinine concentrations (mean ± SEM) in control, after ischemia (DO), and ischemia followed by 1 (D1) or 5 days (D5) of reperfusion

Experimental conditions	Gender	Plasma creatinine concentrations (µmol/L)
Control	Male (n = 4)	40.3 ± 2.4
	Female (n = 4)	31.6 ± 1.7
D0	Male (n = 6)	96.1 ± 5.6
	Female (n = 5)	118.0 ± 6.9
D1	Male (n = 7)	345.0 ± 6.6 *
	Female (n = 6)	200.5 ± 6.6
D5*	Male (n = 3)	511.7 ± 6.4 **
	Female (n = 5)	253.0 ± 6.6

*Five male and three female rats died before day 5. * p <0.05; ** p <0.01 male vs. female

### Effect of ischemia-reperfusion on male sex hormone production

As shown in table 2, hormone plasma levels (estradiol, progesterone and testosterone) did not show significant differences after 60 min of ischemia compared to basal values. After 24 h of reperfusion, estradiol was drastically increased compared to basal values (64.0 ± 9.0 vs. 8.3 ± 0.7 pmol/L, p <0.05) whereas testosterone levels decreased (0.7 ± 0.6 vs. 2.4 ± 0.8 nmol/L, p <0.05). Progesterone levels remained unchanged after 1 day of reperfusion. After 5 days of reperfusion, estradiol increased further up to 336.0 ± 16.8 pmol/L, whereas progesterone levels fell to 4.1 ± 1.8 nmol/L.

**Table 2:** Sex hormone concentrations in blood from male rats (mean ± SEM) in control, after ischemia (D0) and ischemia followed by 1 (D1) or 5 days (D5) of reperfusion

	Estradiol (pmol/L)	Progesterone (nmol/L)	Testosterone (nmol/L)
Control (n = 4)	8,3 ± 0,7	9,2 ± 2.8	2,4 ± 0,8

D0 (n = 6)	7,7 ± 0,8	10,2 ± 3.1	1,7 ± 1.2

D1 (n = 7)	64.0 ± 9.0^a^	8,9 ± 2.9	0,7 ± 0.6^a^

D5 (n = 3)	336.0 ± 16.8^a^	4,1 ± 1.8	1,8 ± 1.7

^a ^p <0.05 compared to D0

### Influence of gender difference on tissue remodeling and cellular proliferation after renal ischemia-reperfusion injury

**Figure 1:**
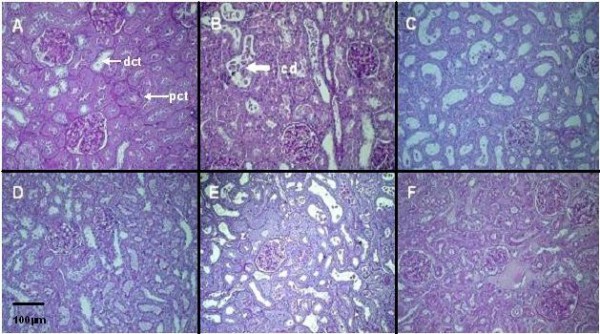
**Renal histological evaluation by light microscopy (Periodic Acid-Schiff, ×400)**. Proximal tubular epithelial cell injury in kidneys from male (A, B, C) and female rats (D, E, F): basal conditions (A and D), after ischemia followed by 1 (B and E) or 5 days of reperfusion (C and F). pct: proximal convoluted tubule; dct: distal convoluted tubule; cd: cell detachment.

**Figure 2:**
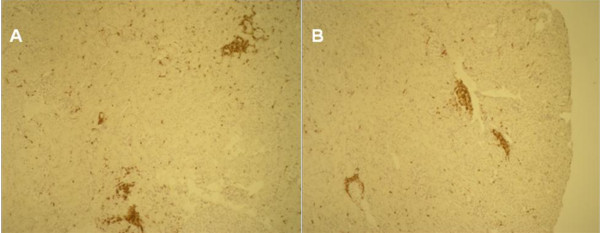
**Interstitial inflammatory changes**: In all times studied, CD3+ T lymphocytes are the major inflammatory cell population. A; Kidney from Rat Male at Day 5. B; Kidney from Female Rat At Day 5. Magnification ×100.

**Figure 3:**
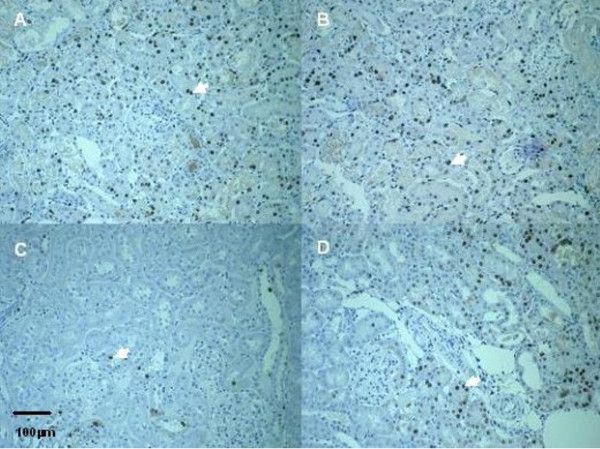
**Representative proximal tubular PCNA staining (arrow)**. Kidneys from male (A, B) and female rats (C, D); after ischemia followed by 1 (A, C) or 5 days of reperfusion (B, D) (Magnification × 200).

**Figure 4:**
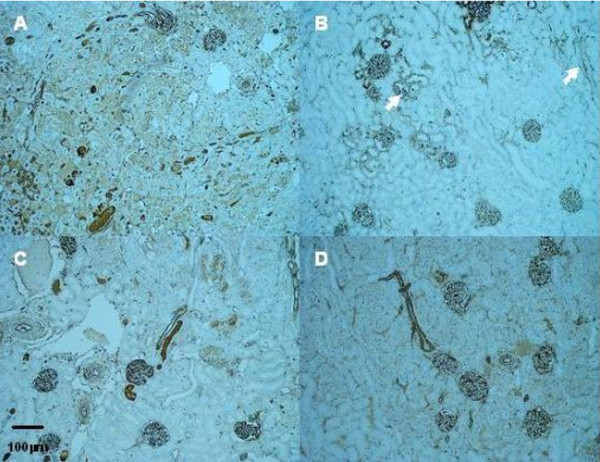
**Representative vimentin expression (arrow)**. Kidneys from male (A, B) and female rats (C, D); after ischemia followed by 1 (A, C) or 5 days of reperfusion (B, D) (Magnification × 200).

**Figure 5:**
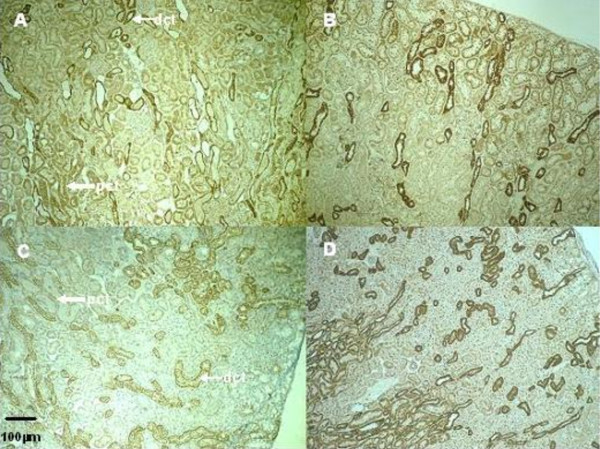
**Representative renal TSPO staining in the outer medulla in kidneys from male (A, B) and female rats (C, D); after ischemia followed by 1 (A, C) or 5 days of reperfusion (B, D) (Magnification ×100)**. At Day 1, Kidneys from male rats exhibited a faint transient cytoplasm staining in proximal tubule epithelial cells. pct: proximal convoluted tubule; dct: distal convoluted tubule.

Warm ischemia with or without 24 h of reperfusion, regardless of gender, induced an extensive tubular injury characterized by tubular necrosis linked to a loss of the apical brush border and intraluminal desquamation. Interestingly, renal lesion levels in males remain high whereas significant improvement was observed in female rats at day 1 or day 5 respectively for cell detachment or loss of brush border determinations (table 3, figure 1). The immunohistochemical studies showed an interstitial infiltration of inflammatory cells after ischemia and during the early reperfusion phase. Infiltrating neutrophils mainly presented at day 1, decreased at day 5 with a drastic increase of CD3 positive lymphocytes (figure 2). These cellular infiltrations at day 5 were significantly attenuated in kidney from female rats compared to males. These results were supported by the PCNA and vimentin immunostaining, which were dramatically increased in male rat kidneys, in comparison to female (table 4, figure 3 and figure 4), indicating a gender effect on regeneration processes. At Day 1, male rat kidneys exhibited a faint transient TSPO staining in proximal tubular epithelial cells (figure 5). In contrast, distal portions of nephrons, including distal tubules and collecting ducts, exhibited a strong cytoplasmic expression and no gender difference was detected (figure 5).

**Table 3:** Semi-quantitative measurement of renal tubular injury, and determination of T lymphocytes (CD3+) and neutrophils (Ly6G+) infiltration per surface areas (10^4 ^µm^2^) in kidneys from male and female rats in control, after ischemia (D0) and ischemia followed by 1 (D1) or 5 days (D5) of reperfusion (mean ± SEM)

Experimental conditions	Control		D0		D1		D5	
Gender	Male	Female	Male	Female	Male	Female	Male	Female
**LBB**	1.0 ± 0.0	1.0 ± 0.0	2.5 ± 0.2	2.5 ± 0.2	3.9 ± 0.1	3.5 ± 0.2	3.0 ± 0.3*	2.0 ± 0.2
**CD**	1.0 ± 0.0	1.0 ± 0.0	1.5 ± 0.2	1.5 ± 0.2	3.1 ± 0.1*	2.2 ± 0.2	2.0 ± 0.3	1.3 ± 0.2
**T CD3+ cells**	2.7 ± 0.3	2.6 ± 0.2	3.4 ± 0.3*	1.6 ± 0.1	1.6 ± 0.2	1.3 ± 0.3	13.3 ± 0.8*	9.8 ± 0.6
**Ly6G+ cells**	0	0	1.2 ± 0.4	0.6 ± 0.3	9.5 ± 0.5*	3.8 ± 0.6	2.1 ± 0.3*	0.8 ± 0.3

LBB: Loss of Brush Border; CD: Cell Detachment, *p <0.05 vs. Female.

**Table 4:** Semi-quantitative evaluation (mean ± SEM) of renal PCNA and vimentin expressions in male and female rats in control, after ischemia (D0) and ischemia followed by 1 (D1) or 5 days (D5) of reperfusion

	Male	Female		
**PCNA index (%)**				
Control	1.5 ± 1.2	0.8 ± 0.9		
D0	5.4 ± 0.7	5.1 ± 0.8		
D1	80.0 ± 3.1*	10.6 ± 1.1		
D5	41.4 ± 2.1*	13.3 ± 1.1		

**Vimentin (%)**				
Control	0	0		
D0	0.3 ± 0.7	0.2 ± 0.6		
D1	14.2 ± 1.7*	5.0 ± 1.3		
D5	9.1 ± 0.8*	2.9 ± 0.6		

*p <0.0001 vs. Female.				

**TSPO**	**Proximal tubule**		**Distal tubule**	

	**Male**	**Female**	**Male**	**Female**
Control	0	0	+++	+++
D0	0	0	+++	+++
D1	+	0	+++	+++
D5	0	0	+++	+++

We used an arbitrary grading scale to assess TSPO expression: 0: No staining; +: Faint staining; ++: Moderate staining; and +++: Strong staining. This staining level is representative of ten high-power fields in each experimental condition.

## Discussion

In uninephrectomized mature rats, renal ischemia-reperfusion resulted in a severe acute renal failure, which affected more predominantly male than female animals as previously reported [11-13,22,23]. In our study, we showed a higher mortality rate for males in comparison to females following 60-min of renal ischemia with contralateral nephrectomy, associated with further deterioration of kidney function. The modulation of systemic male sex hormone levels by renal ischemia-reperfusion could explain male sensitivity to renal injury. Experiments with orchidectomy or ovariectomy as well as with various hormone treatments in male and female animals helped demonstrate the crucial role of sex hormones. Indeed, administration of estradiol in myocardial ischemia-reperfusion induced a rapid recovery of hemodynamic parameters [6,8]. Moreover, exogenous estradiol attenuates the hepatocellular injury induced by ischemia reperfusion in a reduced-size liver mouse model [9]. These results support a protective effect of female sex hormone in several organs subjected to IRI. In male rats, renal IRI was improved by orchidectomy and by estradiol administration, suggesting that either lowering androgen concentrations, or increasing the estradiol-androgen ratio, may have a protective role [11,13]. In contrast, in females, ovariectomy and or testosterone administration did not have any impact on renal function after IRI [11,13]. In addition, it was reported that male sex steroids cause immune suppression, while female sex hormones maintain immune response following trauma-hemorrhage [24]. In the present study, we showed a decrease in testosterone plasma levels after 1 day of reperfusion and a concomitant large increase in estradiol plasma levels. In accordance with our findings, this modulation has also been reported in male rats during experimental septic shock [25] and trauma-hemorrhage shock [26]. The activation of extra-testis aromatase transforming testosterone into estradiol has been found to be involved in experimental septic and hemorrhagic animal models and potentially due to upregulation of aromatase expression likely induced by acute increase in adrenal cortisol production [27]. However, the influence of decreased plasma testosterone and increased estradiol levels on tissue remodelling remains to be elucidated. Following the hypothesis of a detrimental effect of testosterone, and a protective effect of estradiol, we can speculate that our results may be due to compensatory regulatory effects. In addition, the trend towards a decrease in progesterone levels could explain, at least in part, the sensitivity of males to renal IRI.

After ischemia, kidneys from uninephrectomized male and female rats develop, at early reperfusion time points, a similar pattern of histopathological damages, according to previous reports [14]. Importantly, we showed that prolonged reperfusion (5 days) induced more severe tubular damage in kidneys from male compared to female rats. In the past, only few studies, including histological data, have been performed both in male and female rodents [11-13,22,23]. Hu et al. showed that ischemia time necessary to obtain IRI in mice was 35 to 45 min in male and 75 min in female, suggesting increased sensitivity of males to IRI [12]. According to these results, in a limited number of mice, Park et al. showed higher severe proximal tubular damage in males in comparison to females after 30 min of ischemia combined with 24 h of reperfusion [13]. In contrast, using a similar model, Muller et al. failed to show any histological differences between sexes after reperfusion [11]. This discrepancy with our results is likely due to the different ischemia-reperfusion times.

Inflammation is a major component of renal IRI. In our study, kidney from female exhibited a less degree of inflammation as shown by CD3 positive cells and neutrophils recruitment. Previous studies have already demonstrated the role of T lymphocytes as modulators of renal IRI and neutrophils as critical mediators of renal parenchymal injury [15] suggesting in our condition a protection against cellular infiltration related to gender.

Renal tubular epithelial cell dedifferentiation is thought to be a prerequisite for regenerative proliferation after IRI [28]. During the recovery process, surviving kidney cells dedifferentiated to mesenchymal phenotype and then proliferated and migrated to replace lost cells, commonly accepted as an epithelial mesenchymal transition process [29]. Under these conditions, new expression of mesenchymal markers like vimentin occurred in deteriorating and atrophic tubules. Vimentin is an intermediate filament protein expressed only in the mesenchymal cells and has been used as a marker for epithelial mesenchymal transition [30]. In a rat renal vascular occlusion model, Witzgall et al. previously reported that vimentin was detected 1 day post-clamping and prominently expressed 5 days after reperfusion [28]. Concomitantly with vimentin expression, authors described a maximal expression of PCNA 2 days post-ischemia in the proximal tubule [28]. PCNA is a marker for the G1-S transition phase cell cycle and hence mitogenesis. In our study, PCNA and vimentin expression analysis indicated a nephrogenic repair process more pronounced in male rats than in the female rats, related to a more severe injury after reperfusion. These results support a predominant tissue remodelling in male rats related to an extensive renal injury. However, at Day 5, in the female group a decrease in PCNA index and vimentin expression was associated with attenuation of tubular disorders, whereas in males these markers remained elevated in relation to a persistence of major tubular disorders. This underscores gender differences in renal tubular alterations and tissue remodelling after IRI.

There is increasing evidence that mitochondria play a central role in IRI mechanisms [31,32]. Our results supported a previous study in a porcine renal ischemia-reperfusion model, which showed that mitochondrial TSPO protein expression was increased 24h after reperfusion, particularly in the proximal tubule, which does not express this protein constitutively [33]. In addition, we have recently suggested that TSPO integrity is required for renal proximal tubular cell survival [18] and previous studies demonstrated its implication in cell cycle regulation [16,34]. In the present study, TSPO expression was more pronounced in proximal tubules of male rats after 24h of reperfusion, in association with the severity of tubular injury and PCNA staining. The renal distal tubular epithelial cells which express constitutively TSPO, are well adapted to the hypoxic environment of the renal medulla and appear to be more able to withstand hypoxic stress than the epithelium of the proximal tubule [35,36]. The transient expression of TSPO in proximal tubular cells paralleled with PCNA staining results at D1, suggested a transient putative role of TSPO in the mitochondria biogenesis and cell functions at the early time of reperfusion. However, TSPO modulation was not clearly correlated with steroid synthesis. Collectively, these data support the different roles of TSPO depending on its location. In steroid-producing tissues (adrenal glands and gonads), TSPO has been implicated in the regulation of cholesterol transport into the mitochondria, where it is metabolized by CYP11A1 to pregnenolone, the precursor of all steroids. In tissues or organs that are not directly involved in steroidogenesis, TSPO could be involved in a protective effect against lesion-promoting mechanisms like apoptosis and maintain mitochondrial integrity [18].

In conclusion, we showed that male rats were more sensitive to renal IRI than female rats. These gender differences were found in tubular injury development, as well as in the nephrogenic repair process indicated by PCNA and vimentin expression. This sensitivity was associated with modulation of male sex hormone production in response to renal IRI. This adaptive response could be related to an extension of organ failure or a compensatory mechanism to limit renal injury. In addition, TSPO expression was transiently increased in proximal tubules in male rats during IRI process, supporting a putative role of this protein, which will be defined with further studies.

## Competing interests

The authors declare that they have no competing interests.

## Authors' contributions

DAH participated in the design of the study and performed experiments. FF participated in the design of the study and performed experiments and the statistical analysis. JMG performed histopathological analysis, including immunostaining. RR and TH conceived of the study, and participated in its design and coordination. RR, TH and GM helped to draft the manuscript. All authors read and approved the final manuscript.

## References

[B1] Spitzer JA Zhang P Protein tyrosine kinase activity and the influence of gender in phagocytosis and tumor necrosis factor secretion in alveolar macrophages and lung-recruited neutrophils Shock 1996 6 426 433 10.1097/00024382-199612000-00007 8961393

[B2] Miller L Hunt JS Sex steroid hormones and macrophage function Life Sci 1996 59 1 14 10.1016/0024-3205(96)00122-1 8684265

[B3] Angele MK Schwacha MG Ayala A Chaudry IH Effect of gender and sex hormones on immune responses following shock Shock 2000 14 81 90 10.1097/00024382-200014020-00001 10947147

[B4] Spitzer JA Zhang P Gender differences in phagocytic response in the blood and liver, and the generation of cytokine induced neutrophil chemoattractant in the liver of acutely ethanol-intoxicated rats Alcohol Clin Exp Res 1996 20 914 920 10.1111/j.1530-0277.1996.tb05271.x 8865968

[B5] Robert R Spitzer JA Effect of female hormones (17b-estradiol and progesterone) on nitric oxide production by alveolar macrophages in rats Nitric Oxide 1997 1 453 462 10.1006/niox.1997.0157 9466950

[B6] Hale SL Birnbaum Y Kloner RA beta-Estradiol, but not alpha-estradiol, reduced myocardial necrosis in rabbits after ischemia and reperfusion Am Heart J 1996 132 258 262 10.1016/S0002-8703(96)90419-6 8701884

[B7] Squadrito F Altavilla D Squadrito G Campo GM Arlotta M Arcoraci V Minutoli L Serrano M Saitta A Caputi AP 17Beta-oestradiol reduces cardiac leukocyte accumulation in myocardial ischaemia reperfusion injury in rat Eur J Pharmacol 1997 335 185 192 10.1016/S0014-2999(97)01201-6 9369372

[B8] Zhai P Eurell TE Cotthaus R Jeffery EH Bahr JM Gross DR Effect of estrogen on global myocardial ischemia-reperfusion injury in female rats Am J Physiol Heart Circ Physiol 2000 279 H2766 2775 11087231 10.1152/ajpheart.2000.279.6.H2766

[B9] Harada H Pavlick KP Hines IN Lefer DJ Hoffman JM Bharwani S Wolf RE Grisham MB Sexual dimorphism in reduced-size liver ischemia and reperfusion injury in mice: role of endothelial cell nitric oxide synthase Proc Natl Acad Sci 2003 100 739 744 10.1073/pnas.0235680100 12522262 PMC141066

[B10] Eckhoff DE Bilbao G Frenette L Thompson JA Contreras JL 17-Beta-estradiol protects the liver against warm ischemia/reperfusion injury and is associated with increased serum nitric oxide and decreased tumor necrosis factor-alpha Surgery 2002 132 302 309 10.1067/msy.2002.125718 12219027

[B11] Muller V Losonczy G Heemann U Vannay A Fekete A Reusz G Tulassay T Szabo AJ Sexual dimorphism in renal ischemia-reperfusion injury in rats: possible role of endothelin Kidney Int 2002 62 1364 1371 10.1111/j.1523-1755.2002.kid590.x 12234307

[B12] Hu H Wong G Batteux F Nicco C Gender difference in the susceptibility to renal ischemia-reperfusion injury in BALB/c mice Tohoku J Exp Med 2009 218 325 329 10.1620/tjem.218.325 19638737

[B13] Park KM Kim JI Bonventre AJ Bonventre JV Testosterone is responsible for enhanced susceptibility of males to ischemic renal injury J Biol Chem 2004 279 52282 5292 10.1074/jbc.M407629200 15358759

[B14] Godet C Goujon JM Petit I Lecron JC Hauet T Mauco G Carretier M Robert Endotoxin-tolerance decreases Ischemia-Reperfusion renal injury in rats: potential role of Interleukin-10 Shock 2006 25 384 388 10.1097/01.shk.0000209528.35743.54 16670641

[B15] Jayle C Favreau F Zhang K Doucet C Goujon JM Hebrard W Carretier M Eugene M Mauco G Tillement JP Hauet Comparison of protective effects of trimetazidine against experimental warm ischemia of different durations: early and long-term effects in a pig kidney model J Physiol Renal Physiol 2007 292 F1082 1093 10.1152/ajprenal.00338.2006 17341718

[B16] Papadopoulos V Baraldi M Guilarte TR Knudsen TB Lacapère JJ Lindermann P Norenberg MD Nutt D Weizman A Zhang MR Gavish M Translocator protein (18kDA): new nomenclature for the peripheral-type benzodiazepine receptor based on its structure and molecular function Trends Pharmacol Sci 2006 27 402 409 10.1016/j.tips.2006.06.005 16822554

[B17] Lacapere JJ Papadopoulos V Peripheral-type benzodiazepine receptor: structure and function of a cholesterol-binding protein in steroid and bile acid biosynthesis Steroids 2003 68 569 585 10.1016/S0039-128X(03)00101-6 12957662

[B18] Favreau F Rossard L Zhang K Desurmont T Manguy E Belliard A Fabre S Liu J Han Z Thuillier R Papadopoulos V Hauet T Expression and modulation of translocator protein and its partners by hypoxia reoxygenation or ischemia and reperfusion in porcine renal models Am J Renal Physiol 2009 297 F177 F190 10.1152/ajprenal.90422.2008 PMC2711711 19386723

[B19] Hauet T Han Z Wang Y Hameury F Jayle C Gibelin H Goujon JM Eugene M Papadopoulos V Modulation of peripheral-type benzodiazepine receptor levels in a reperfusion injury pig kidney-graft model Transplantation 2002 74 1507 1515 10.1097/00007890-200212150-00006 12490782

[B20] Goujon JM Hauet T Menet E Levillain P Babin P Carretier M Histological evaluation of proximal tubule cell injury in isolated perfused pig kidneys exposed to cold ischemia J Surg Res 1999 82 228 233 10.1006/jsre.1998.5526 10090834

[B21] Wright G Reichenbecher V The effect of superoxide and the peripheral benzodiazepine receptor ligands on the mitochondrial processing of manganese-dependent superoxide dismutase Exp Cell Res 1999 246 443 450 10.1006/excr.1998.4331 9925760

[B22] Fekete A Vannay A Ver A Vasarhelyi B Muller V Ouyang N Reusz G Tulassay T Szabo AJ Sex difference in the alteration of Na+, K+ -ATPase following ischemia-reperfusion injury in the rat kidney J Physiol 2003 555 2 471 480 10.1113/jphysiol.2003.054825 14673189 PMC1664838

[B23] Takayama J Takaoka M Sugino Y Yamamoto Y Ohkita M Matsumura Y Sex Difference in Ischemic Acute Renal Failure in Rats: Approach by Proteomic Analysis Biol Pharm Bull 2007 30 1905 1912 10.1248/bpb.30.1905 17917260

[B24] Choudhry MA Bland KI Chaudry IH Vincent JL Berlin, Heidelberg Insight into the mechanism of gender-specific response to trauma-hemorrhage Yearbook of intensive care and emergency medicine 2007 New-York: Springer -Verlag 869 879

[B25] Christeff N Auclair MC Thobie N Fertil B Carli A Nunez EA Effect of estradiol on endotoxin-induced changes in steroid hormone levels and lethality in male rats Circ Shock 1994 44 154 159 7600639

[B26] Choudhry MA Bland KI Chaudry IH Trauma and immune response-effect of gender differences Injury 2007 38 1382 1391 10.1016/j.injury.2007.09.027 18048037 PMC2692838

[B27] Schneider CP Nickel EA Samy TS Schwacha MG Cioffi WG Bland KI Chaudry IH The aromatase inhibitor, 4-hydroxyandrostenedione, restores immune responses following trauma-hemorrhage in males and decreases mortality from subsequent sepsis Shock 2000 14 347 353 10.1097/00024382-200014030-00019 11028555

[B28] Witzgall R Brown D Schwartz C Bonventre JV Localization of proliferating cell nuclear antigen, vimentin, c-Fos, and clusterin in the postischemic kidney. Evidence for a heterogenous genetic response among nephron segments, and a large pool of mitotically active and dedifferentiated cell J Clin Invest 1994 93 2175 2188 10.1172/JCI117214 7910173 PMC294357

[B29] Bonventre JV Weinberg JM Recent advances in the pathophysiology of ischemic acute renal failure J Am Soc Nephrol 2003 8 2199 2210 10.1097/01.asn.0000079785.13922.f6 12874476

[B30] Vongwiwatana A Tasanarong A Rayner DC Melk A Halloran PF Epithelial to mesenchymal transition during late deterioration of human kidney transplants: the role of tubular cells in fibrogenesis Am J Transplant 2005 5 1367 1374 10.1111/j.1600-6143.2005.00843.x 15888043

[B31] Jassem W Fuggle SV Rela M Koo DD Heaton ND The role of mitochondria in ischemia/reperfusion injury Transplantation 2002 73 493 499 10.1097/00007890-200202270-00001 11889418

[B32] Benitez-Bribiesca L Gomez-Camarillo M Castellanos-Juarez E Mravko E Sanchez-Suarez P Morphologic, biochemical and molecular mitochondrial changes during reperfusion phase following brief renal ischemia Ann N Y Acad Sci 2000 926 165 179 11193033 10.1111/j.1749-6632.2000.tb05610.x

[B33] Zhang K Desurmont T Goujon JM Favreau F Cau J Deretz S Mauco G Carretier M Papadopoulos V Hauet T Modulation of peripheral-type benzodiazepine receptor during ischemia reperfusion injury in a pig kidney model: a new partner of leukaemia inhibitory factor in tubular regeneration J Am Coll Surg 2006 203 353 364 10.1016/j.jamcollsurg.2006.05.012 16931308

[B34] Corsi L Geminiani E Baraldi M Peripheral benzodiazepine receptors in hepatic encephalopathy Curr Clin Pharmacol 2008 3 38 45 10.2174/157488408783329878 18690876

[B35] Gobe GC Johnson DW Distal tubular epithelial cells of the kidney: Potential support for proximal tubular cell survival after renal injury Int J Biochem cell Biol 2007 39 1551 1561 10.1016/j.biocel.2007.04.025 17590379

[B36] Neuhofer W Beck FX Cell survival in the hostile environment of the renal medulla Annu Rev Physiol 2005 67 531 555 10.1146/annurev.physiol.67.031103.154456 15709969

